# Chemical and Biological Investigation of the Endophytic *Aspergillus terreus* (SU5) Inhabiting Date Fruits (*Phoenix dactylifera*)

**DOI:** 10.3390/jof12040249

**Published:** 2026-03-30

**Authors:** Ahmed Abdel-Hadi, Mohammed Alaidarous, Abdulrahman Alatiq, Yahya Madkhali, Saeed Banawas, Mohamed Abouelela, Abdallah Hassane

**Affiliations:** 1 Department of Medical Laboratory Sciences, College of Applied Medical Sciences, Majmaah University, Majmaah 11952, Saudi Arabia; m.alaidarous@mu.edu.sa (M.A.); a.alatiq@mu.edu.sa (A.A.); y.madkhali@mu.edu.sa (Y.M.); s.banawas@mu.edu.sa (S.B.); 2 Department of Pharmacognosy, Faculty of Pharmacy (Boys), Al-Azhar University, Cairo 11884, Egypt; m_abouelela@azhar.edu.eg; 3 Department of Botany and Microbiology, Faculty of Science, Al-Azhar University, Assiut Branch, Assiut 71524, Egypt; abdallahhassane@azhar.edu.eg

**Keywords:** *Aspergillus terreus*, endophytic fungi, date fruits, *Phoenix dactylifera*, secondary metabolites, antioxidant activity, cholinesterase inhibition, cytotoxity

## Abstract

Endophytic fungi associated with date fruits (*Phoenix dactylifera*) are mostly under-explored, despite their potential as reservoirs of natural compounds. The aims of this study were to characterize the endophytic fungus *Aspergillus terreus* (SU5) isolated from date fruits, and to investigate its biological activities and chemical profile for the first time. Morphological and molecular methods were utilized to identify *Aspergillus terreus*. A liquid chromatography–mass spectrometry analysis (LC/MS/MS) was conducted to determine the chemical profile of the crude extract. Biological properties were investigated through acetylcholine esterase and butyrylcholine esterase inhibition, cytotoxicity assays against MCF-7 and MCF-7/Adr, and antioxidant assays. LC/MS/MS of the fungal extract resulted in the detection of 39 of established secondary metabolites, primarily comprising polyketides, quinones, and phenolic derivatives. The crude extract demonstrated significant antioxidant activity, especially in the ABTS assay (IC_50_ = 50.18 μg/mL), considerable cytotoxicity against MCF-7 breast cancer cells, diminished efficacy against the drug-resistant MCF-7/Adr cell line, and preferential inhibition of butyrylcholinesterase compared to acetylcholinesterase. While none of the identified compounds are novel, numerous metabolites are documented here for the first time from an endophytic *A. terreus* associated with date fruits. The findings underscore date fruits as a prospective ecological niche for a chemically varied endophytic fungus with potential pharmaceutical significance.

## 1. Introduction

Fungi produce a wide range of secondary metabolites that possess significant biological activity, many of which can be utilized in medicine, agriculture, and biotechnology. Among them, endophytic fungi are attracting growing interest as valuable sources of bioactive natural products, as they reside symbiotically within plant tissues without causing harm to the host [1,2]. The unique ecological relationships between endophytes and their host plants frequently trigger the synthesis of metabolites that are distinct from those produced by free-living or pathogenic fungi, which makes endophytic fungi a significant source of chemical variation [3].

The date palm (*Phoenix dactylifera* L.) is the most extensively farmed crop in the Middle East and North Africa. It is utilized extensively and has a significant history of use in traditional medicine. The fruit is rich in nutrients, including dietary fiber, carbohydrates, proteins, minerals, vitamins, flavonoids, and phenolic components which may influence the metabolic profiles of associated endophytic microorganisms [4]. Hamad et al. [5] demonstrated that the Saudi date fruit varieties (Helwat Al Jouf, Al Sagey, and Al Sour) exhibit substantial antioxidant activity, primarily due to their phenolic compounds and flavonoids. Al Sagey cultivar demonstrated the highest antioxidant capabilities and the most substantial phenolic contents. Al Sagey and Helwat Al Jouf displayed analogous glutathione and ascorbate redox statuses, but Al Sour exhibited the lowest glutathione redox state. Despite the long-standing agricultural and cultural importance of date palms, their endophytic fungal communities—particularly those inhabiting date fruits—remain poorly explored from a chemical and biological perspective. Mahmoud et al. [6] examined the community of endophytic root fungi in date palms from three plantations located in Adrar Province (Southeast Algeria). The predominant fungal endophytes were *Fusarium* sp. (30.9%), followed by *Aspergillus terreus* (15.4%). Four isolates were evaluated for their capacity to promote wheat growth and the production of secondary metabolites [6]. Two fungi, namely *Penicillium citrinum* TDPEF34, and *Geotrichum candidum* TDPEF20, originating from healthy and brittle leaf diseased (BLD) date palm root microbiomes, respectively, were investigated for their potential to serve as cell factories for the production of bioactive secondary metabolites. Extracts from both endophytic fungi demonstrated a high content of polyphenols and flavonoids and, consequently, exhibited potent antioxidant activity [7]. Piombo et al. [8] used metagenomic analysis to compare microbial inhabitants in the pulp and peel of “Medjool” dates at different stages of fruit development. In all fruit sections, *Penicillium*, *Cladosporium*, *Aspergillus*, and *Alternaria* were the most common genera, but taxonomic distribution varied by time and tissue type. While *Penicillium* was more numerous in the pulp during green growth (Kimri), *Aspergillus* was more common in the peel during brown growth (Tamer).

Recent studies highlight *A. terreus* as a prolific source of structurally diverse secondary metabolites including polyketides, quinones, phenolic derivatives, and butenolides which have been isolated from terrestrial, marine, and endophytic strains [9,10]. Advanced analytical approaches such as LC-MS-based metabolomics, high-resolution mass spectrometry, and NMR spectroscopy have facilitated the identification and structural characterization of these metabolites [11]. Many of the newly discovered compounds have demonstrated significant biological activities including antimicrobial, cytotoxic, phytotoxic, and enzyme inhibitory effects [12,13]. In particular, terrein derivatives, sulfur-containing metabolites, and polyketide-derived compounds have attracted attention due to their potential pharmacological relevance [14,15]. Overall, these studies emphasize the metabolic diversity of *A. terreus* and support its importance as a promising source of bioactive natural products, while also highlighting the utility of metabolomics and modern natural product discovery tools [16].

Oxidative stress and enzyme dysregulation are key factors involved in the pathogenesis of several chronic diseases, including neurodegenerative disorders and cancer. Antioxidants play a critical role in mitigating oxidative damage [17], while cholinesterase inhibitors—particularly butyrylcholinesterase (BChE) inhibitors—are of growing interest due to the increased role of BChE in the progression of Alzheimer’s disease [18]. In parallel, the search for new anticancer agents remains a major focus of natural product research, and compounds capable of overcoming or bypassing multidrug resistance mechanisms are particularly highly sought. Fungal secondary metabolites, notably phenolic and quinone-containing compounds, have been reported to exhibit antioxidant, cholinesterase inhibitory, and cytotoxic activities, supporting the continued exploration of fungal sources for such bioactivities [19]. Hence, the objectives of this work were to (i) isolate and identify secondary metabolites produced by this endophytic *A. terreus* isolated from date fruits (*Phoenix dactylifera*), and (ii) evaluate the antioxidant, cholinesterase inhibitory, and cytotoxic activities of the fungal extract.

## 2. Materials and Methods

### 2.1. Isolation, Purification and Characterization of Endophytic Fungus

Fresh date samples from a popular variety (Sukkari) were collected from farms of Zulfi City, Saudi Arabia. The samples were aseptically collected in sterile plastic bags (3M Company, Maplewood, MN, USA), transported to the laboratory within four hours, and maintained at 4 °C until fungal analysis. Surface sterilization of collected samples was performed by immersing them in 70% ethanol for 5 min, followed by 5% sodium hypochlorite for an additional 5 min, and finally rinsing with sterile distilled water [20]. Sterilized dates were cut to small fragments by using a sterile scalpel and forceps, and four fragments were placed on Potato Dextrose Agar medium (PDA) containing antibiotics (streptomycin and chloramphenicol) and incubated at 28 °C for 5–7 days. The grown colonies were purified by repeated subculturing on fresh PDA plates using the hyphal tip isolation method to obtain pure cultures. This process was repeated until morphologically uniform colonies were achieved. Fungal strain was identified based on micro- and macromorphological diagnostic characteristics in culture, combined with subsequent molecular methods [21]. DNA was isolated from a pure culture of the fungal strain using Qiagen kits based on the manufacturer’s instructions (QIAGEN, Valencia, CA, USA). The universal primers ITS1 (5′-TCCGTAGGTGAACCTGCG-3′) and ITS4 (5′-TCCTCCGCTTATTGATATGC-3′) were employed to amplify the ITS regions of the fungal strain. PCR amplification was performed in a total reaction volume of 25 mL, utilizing 1× PCR buffer (DreamTaq™) in a C1000TM Thermo Cycler (Bio-Rad Laboratories GmbH, Feldkirchen, Germany). The initial denaturation phase occurred at 94 °C for 5 min followed by 35 cycles including denaturation at 94 °C for 30 s, annealing at 49 °C for 30 s, and extension at 72 °C for 1 min [22]. The last extension phase at 72 °C for 5 min was performed following the preceding 35 cycles. The DNA amplicons were sequenced utilizing the Gene Analyzer 3121 sequencer with the same primers, ITS1 and ITS4 (Macrogen Co., Seoul, South Korea). The ITS sequences were examined with BioEdit version 7.2.5. The isolate was identified by comparing sequencing data against databases with the BLAST tool from the GenBank database (http://www.ncbi.nlm.nih.gov/BLAST/, accessed on 23 March 2026).

### 2.2. Fermentation and Extraction of Fungal Metabolites

Fermentation of the isolated fungus was performed utilizing solid state fermentation [21] on 100 g autoclaved rice and 100 mL distilled water in 1 L Erlenmeyer flasks. After cooling, the flasks were inoculated with 2 mL spore suspension (10^5^ cfu/mL) and incubated at 28 ± 2 °C for thirty days. After that, the fermented rice was subjected to extraction of fungal metabolites using ethyl acetate (Analytical Grade, Alpha Chemika, Mumbai, India) for 24 h at room temperature [21]. The extract was filtered, dried with a rotavapor (BÜCHI R-114, Flawil, Switzerland), and kept at −4 °C for further work.

### 2.3. Estimation of Total Phenolic Content

Total phenolic content determination was assessed using the modified method of Suleria et al. [23]. A half mL of the extract (10 mg/mL) was mixed with the same volume of Folin–Ciocalteu phenol reagent, followed by 1 mL of 10% Na_2_CO_3_ (AL-Nasr Chemicals Co., Cairo, Egypt), and then the mixture was incubated under shaking at 180 rpm for 60 min at 25 °C in the dark. Measurement of the absorbance was carried out at 750 nm. Phenolic content was expressed as gallic acid equivalent (GAE) (mg/g) through the standard curve equation: y = 0.0169x–0.1172, R^2^ = 0.9588. The standard curve of gallic acid was linear between 0.5 and 100 μg/mL.

### 2.4. Determination of Total Flavonoid Content

The measurement of total flavonoid content was performed following the method reported by Nicolescu et al. [24]. A 0.5 mL quantity of the extract (10 mg/mL) was carefully added to 1.0 mL of a 2% (*v*/*v*) AlCl_3_·6H_2_O (AL-Nasr Chemicals Co., Cairo, Egypt) ethanolic solution and the absorbance was measured at 430 nm after 10 min. Total flavonoid content was expressed as quercetin equivalent (QE) (mg/g) by employing the standard curve equation: y = 0.0208x–0.2381, R^2^ = 0.9678. The calibration curve of quercetin (Sigma-Aldrich Chemie GmbH, Taufkirchen, Germany) was linear between 0.5 and 100 μg/mL.

### 2.5. Antioxidant Activity Assays

#### 2.5.1. DPPH Scavenging Activity Assay

Briefly, 1.8 mL of 0.1 mM DPPH (1,1-Diphenyl-2-picryl hydrazyl) (Sigma-Aldrich, Germany) (4 mg/100 mL of methanol) solution was added to 0.2 mL of the fungal extract in absolute methanol at various concentrations (1, 0.8, 0.6, 0.4, 0.2, 0.1 and 0.05 mg/mL) in addition to the blank. Absorbance was determined by a spectrophotometer ( Bibby Scientific Ltd., Stone, Staffordshire, UK) at 517 nm after 30 min [25]. Butylated hydroxytoluene (BHT) (Chemical Industries Development Company, Cairo, Egypt) was employed as a positive control at concentrations ranging between 100 and 10 μg/mL. The following formula was employed to determine the capacity to scavenge the DPPH radical:

% DPPH radical scavenging = (A0–A1)/A0 × 100

where A0 is the negative control absorbance (methanol (AL-Nasr Chemicals Co., Cairo, Egypt) and DPPH) and A1 is the sample absorbance (DPPH, methanol and sample). The IC_50_ was calculated as IC_50_ = (50–b)/a, obtained by interpolation from linear regression analysis where “a” represents the slope and “b” is the Y-intercept.

#### 2.5.2. ABTS Scavenging Assay

The fungal extract was dissolved in dimethyl sulfoxide (DMSO) (MilliporeSigma—St. Louis, MI, USA) and diluted with methanol to a concentration of 10 mg/mL. Subsequently, five serial dilutions were prepared at final concentrations of 7.81, 15.625, 31.25, 62.5, and 125 μg/mL. The assay was conducted in microplates in accordance with the method of Arnao et al. [26], which was subsequently adopted by Elkholy et al. [27]. Briefly, 192 mg of 2,2′-azino-bis(3-ethylbenzothiazoline-6-sulphonic acid) (ABTS) (Sigma-Aldrich, Germany) was dissolved in distilled water and transferred to a 50 mL volumetric flask. The volume was subsequently topped up with distilled water. Then, 17 μL of 140 mM potassium persulphate was combined with 1 mL of the previous solution, and the mixture was incubated in the dark for 24 h. The final ABTS dilution used in the assay was obtained by making 1 mL of the reaction mixture up to 50 mL with methanol. The reaction was incubated at room temperature for 30 min in the dark following the addition of 10 μL of the fungal extract to 190 μL of the freshly prepared ABTS reagent in a 96-well plate. A microplate reader (BMG LABTECH GmbH, Ortenberg, Germany) was employed to measure the decrease in ABTS color intensity at 734 nm at the conclusion of the incubation period. To serve as a standard positive control, Trolox (Sigma-Aldrich, Germany) was prepared at the following final concentrations: 8.75, 6.25, 5, 3.75, and 2.5 μg/mL. The scavenging capacity was determined using the following equation:

Percentage inhibition = ((Absorbance of blank − Absorbance of the test)/(Absorbance of blank)) × 100

After that, the IC_50_ value was calculated.

#### 2.5.3. Metal Chelation Assay

The fungal extract was dissolved in DMSO and subsequently diluted with methanol to a concentration of 10 mg/mL. Subsequently, five serial dilutions were prepared at concentrations of 4000, 3500, 2500, 1500, and 1000 μg/mL, resulting in final concentrations of 333.33, 500, 833.33, 1166.67, and 1333.33 μg/mL. The assay was conducted in microplates in accordance with the method of Santos et al. [28], with minimal modifications. To prepare the 96-well plate, 20 μL of the freshly prepared ferrous sulphate (Alpha Chemika, Mumbai, India) (0.3 mM) was combined with 50 μL of the fungal extract and 50 μL of acetate buffer with a pH of 6. This was followed by the addition of 30 μL of ferrozine (Sigma-Aldrich, Germany) (0.8 mM) to each well. The reaction mixture was incubated at ambient temperature for a duration of 10 min. The microplate reader was employed to measure the decrease in color intensity at 562 nm at the conclusion of the incubation period. Five serial dilutions were prepared at concentrations of 29.224, 14.612, 10.959, 7.306, and 5.8448 μg/mL from a 292.24 μg/mL EDTA (Alpha Chemika, Mumbai, India) stock solution in water. The chelation capacity was determined using the following equation:

Percentage inhibition = ((Absorbance of blank − Absorbance of the test)/(Absorbance of blank)) × 100

After that, the IC_50_ value was calculated.

#### 2.5.4. ORAC Assay

The following dilutions were prepared, 1000, 800, 600, 500, 400, 200, 100, and 50 μM, all of which were derived from a 2 mM Trolox stock solution in MeOH. The fungal extract was dissolved in 50% methanol and subsequently diluted with methanol to a concentration of 40 μg/mL. The assay was conducted in accordance with the method of Liang et al. [29], with modifications made by Elkholy et al. [27]. In brief, 10 μL of the prepared sample was incubated with 30 μL of fluoresceine (Sigma-Aldrich, Germany) (100 nM) at 37 °C for 10 min. For background measurement, fluorescence measurements were conducted for three cycles (cycle time, 90 s) at 485 EX and 520 EM nm. Subsequently, 70 μL of freshly prepared 2,2′-Azobis(2-amidinopropane) dihydrochloride (AAPH) (Sigma-Aldrich, Germany) (240 mM) was promptly added to each well. The fluorescence measurement (485 EX, 520 EM, nm) was maintained for 60 min, with a maximum of 40 cycles, each lasting 90 s. μM Trolox equivalents were used to compute the antioxidant effect of the fungal extract through substitution in the linear regression equation:

y = 3526.176x + 527,521.245; R^2^ = 0.997

### 2.6. The Brine Shrimp Cytotoxicity Test

The brine shrimp cytotoxicity test (BSCT) is a method used to determine the cytotoxicity of the fungal extract to brine shrimp larvae. The extract was dissolved in DMSO at varying concentrations (100–2.5 mg/mL). Then, 100 μL of each extract concentration was added to vials containing 5 mL of seawater and 10 brine shrimp larvae. After 24 h, the number of viable larvae in each vial was counted. DMSO was used as a negative control, and the percentage mortality was determined. The concentration of the extract that killed 50% of the larvae (LC_50_) was calculated using probit analysis [30].

### 2.7. Cell Culture Cytotoxicity Assay

MCF-7 (Breast Adenocarcinoma) and MCF-7-Adr (Doxorubicin-resistant Breast Cancer) were purchased from Nawah Scientific Inc., located in Mokatam, Cairo, Egypt.

Dulbecco’s Modified Eagle’s Medium (DMEM) (Sigma-Aldrich, Germany) supplemented with 100 mg/mL of streptomycin, 100 units/mL of penicillin, and 10% of heat-inactivated fetal bovine serum was used to maintain the cells at 37 °C in a humidified 5% (*v*/*v*) CO_2_ atmosphere. The SRB assay was used to evaluate the viability of the cells. Aliquots of 100 μL cell suspension (5 × 10^3^ cells) were added to 96-well plates and incubated in complete media for 24 h. The cells were treated with an additional aliquot of 100 μL medium, which contained fungal extract at varying concentrations (100, 10, 1, 0.1, and 0.01 μg/mL). Doxorubicin was produced as a standard positive control at varying concentrations. The cells were fixed by replacing the media with 150 μL of 10% trichloroacetic acid (TCA) and incubating at 4 °C for 1 h after their exposure to the extract. The cells were rinsed five times with distilled water after the TCA solution was removed. Aliquots of 70 μL Sulforhodamine B (SRB) (Sigma-Aldrich, Germany) solution (0.4% *w*/*v*) were added and incubated at room temperature for 10 min in a dark place. Plates were rinsed three times with 1% acetic acid and allowed to air-dry overnight. Then, 150 μL of TRIS (10 mM) was added to dissolve the protein-bound SRB stain. The absorbance was measured at 540 nm using an Infinite F50 microplate reader (Tecan Group Ltd., Männedorf, Switzerland), and the IC_50_ value was calculated [31].

### 2.8. Acetylcholine Esterase and Butyrylcholine Esterase Inhibition Assay

The samples were initially dissolved in DMSO and subsequently diluted to the desired concentrations with distilled water. The resulting fungal extracts were prepared at the following final concentrations: 500, 250, 125, 62.5, and 50 μg/mL. The final concentrations of standard donepezil in methanol were 1, 0.1, 0.01, 0.001, and 0.0001 μM. These concentrations are equivalent to 0.416, 0.0416, 0.00416, 0.000416 and 0.0000416 μg/mL. The enzyme acetylcholinesterase (Sigma-Aldrich, Germany) with its substrate acetylthiocholine iodide, the enzyme butyrylcholineesterase with its substrate butyrythiolcholine iodide, and the indicator (DTNB Ellman’s reagent) (Sigma-Aldrich, Germany) were utilized in the following assay. The assay was carried out according to the method of Osman et al. [32] with minor modifications. In brief, 10 μL of the indicator solution (0.4 mM in buffer (1): 100 mM tris buffer, pH 7.5) was added to a 96-well plate, followed by 20 μL of enzyme solution (acetylcholine esterase or butyrylcholine esterase at a final concentration of 0.02 U/mL in buffer (2): 50 mM tris buffer, pH 7.5, containing 0.1% bovine serum albumin). Subsequently, 20 μL of the fungal extract solution was introduced, followed by the addition of 140 μL of buffer (1). The mixture was allowed to sit for 15 min at room temperature. Subsequently, 10 μL of the substrate (0.4 mM acetylcholine iodide buffer (1)) was immediately added to all wells. The plate was incubated in a dark place for 20 min at room temperature. At the end of the incubation time, the color was assessed at 412 nm with the FluoStar Omega microplate reader, and the IC_50_ value was calculated.

### 2.9. Liquid Chromatography–Mass Spectrometry Analysis (LC/MS/MS)

The sample was analyzed using liquid chromatography–electrospray ionization–tandem mass spectrometry (LC-ESI-MS/MS) (Agilent Technologies, Waldbronn, Germany) with an Exion LC AC system for separation and a SCIEX Triple Quad 5500+ MS/MS system fitted with electrospray ionization (ESI) for detection. The separation was conducted using an Ascentis^®^ C18 column (4.6 × 150 mm, 3 µm) (Merck-Sigma group, Schnelldorf, Germany). The mobile phases were composed of two eluents: A consisted of 0.1% formic acid and B consisted of acetonitrile (LC grade). The mobile phase gradient was programmed as follows: 10% B at 0–1 min, 10–90% B from 1 to 33 min, 90% B from 33 to 37 min, and 10% B from 37.1 to 40 min. The injection volume was 10 µL, and the flow rate was 0.7 mL/min. The scan (EMS-IDA-EPI) was conducted in negative ionization mode for MS/MS analysis [33]. The identification of secondary metabolites was performed using a LC–ESI–MS/MS SCIEX Triple Quad 5500+ MS/MS system for putative dereplication, which is low resolution mass measurement, so tentative identification could be made of the compounds based on both detected mass value and fragmentation patterns comparison with *Aspergillus* compound database, literature reports and established databases prediction libraries [34,35].

### 2.10. Data Analysis

Three replicates of each experiment were implemented. The data were analyzed and established using Microsoft Excel^®^ and SPSS software, version 16, and presented as the mean ± SD. Data were established by analysis of variance (one-way ANOVA) as being below the 0.05 level of significance.

## 3. Results

### 3.1. Identification of Fungal Strain

**Figure 1:**
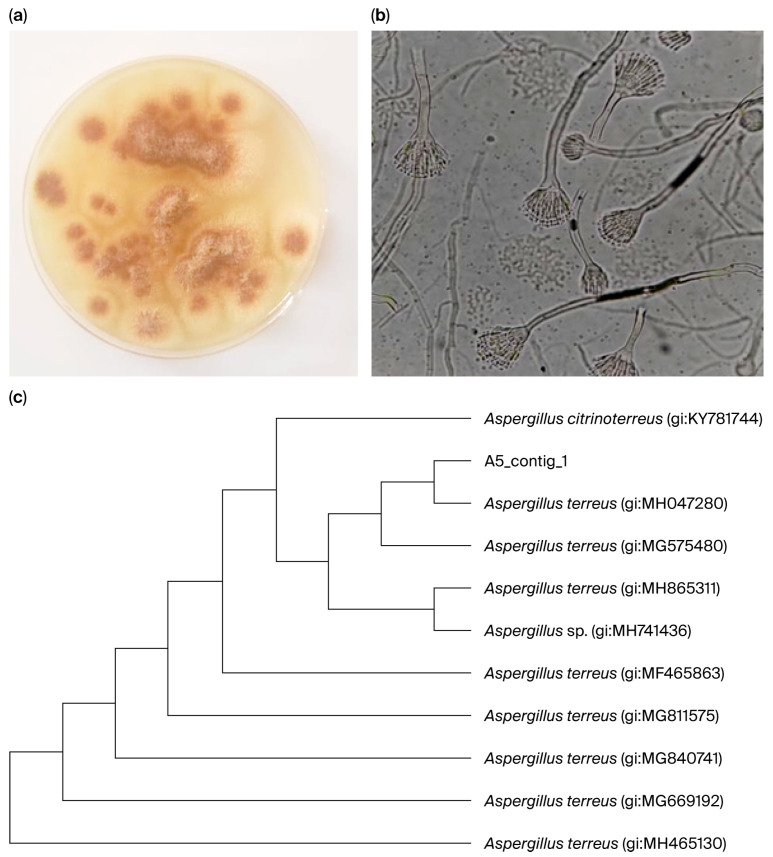
Characteristics of fungal strain isolated from Sukkari dates fruits: (**a**) cultural morphology; (**b**) microscopic image; (**c**) phylogenetic tree.

The current investigation utilized an endophytic fungal strain obtained from Sukkari date fruits. This strain was identified using morphological and molecular techniques. The fungal colony cultivated on PDA exhibited rapid growth, characterized by a cinnamon-brown color and a rough texture with cottony mycelium. Microscopic examination revealed elongated, colorless conidiophores with biserrate conidia (Figure 1). High-quality DNA isolated from endophytic fungus utilized ITS1 and ITS4 as primers for PCR. The genomic DNA of the successfully amplified fungus measured 634 bp in length. The results indicated a fungus strain with almost 99% similarity to *Aspergillus terreus* (gi:MH047280). The fungus strain was designated *A. terreus* (SU-5) with accession number MH047280.1 (Supplementary Material Table S1).

### 3.2. Total Phenolic and Flavonoid Content and Antioxidant Activities of A. terreus Extract

Table 1 presents the total concentrations of phenolics and flavonoids in endophytic *A. terreus* during solid-state fermentation, revealing a phenolic content of 93.13 mg GAE/g and a flavonoid content of 22.70 mg QE/g. The EtOAc extract of *A. terreus* exhibited a DPPH IC_50_ value of 575.64 μg/mL, but the IC_50_ for ABTS radical scavenging activity was reported as 50.18 μg/mL. The fungal extract’s chelating activity was evaluated, showing an IC_50_ value of 1007.82 μg/mL for ferrous ion chelation. The evaluated fungal extract demonstrated an antioxidant scavenging activity of 3879.42 µmol TE/g against peroxyl radicals in the ORAC testing. The results for BHT, Trolox, and EDTA, serving as standard positive controls in several antioxidant activity assays, are presented in Table 1.

### 3.3. Anticancer Activities of A. terreus Extract

The ethyl acetate extract of *A. terreus* was evaluated for its effect on the viability of two cancer cell lines, MCF-7 and MCF-7-Adr, using the SRB assay. The IC_50_ values were determined from dose–response curves, producing 424.73 μg/mL for MCF-7 and 1140.77 μg/mL for MCF-7-Adr, in comparison to doxorubicin. The Artemia cytotoxicity assay of the fungal extract demonstrated an LC_50_ value of 1402.92 µg/mL (Table 2).

### 3.4. Effect of A. terreus Extract on the Inhibition of Acetylcholine and Butyrylcholine Esterases

The results presented in Table 3 indicated that, in comparison with standard donepezil, the EtOAc extract of endophytic *A. terreus* exhibited IC_50_ values of 456.90 and 157.10 μg/mL for the inhibition of acetylcholinesterase and butyrylcholinesterase, respectively.

### 3.5. LC-ESI-MS/MS Analysis of A. terreus Metabolites

**Figure 2:**
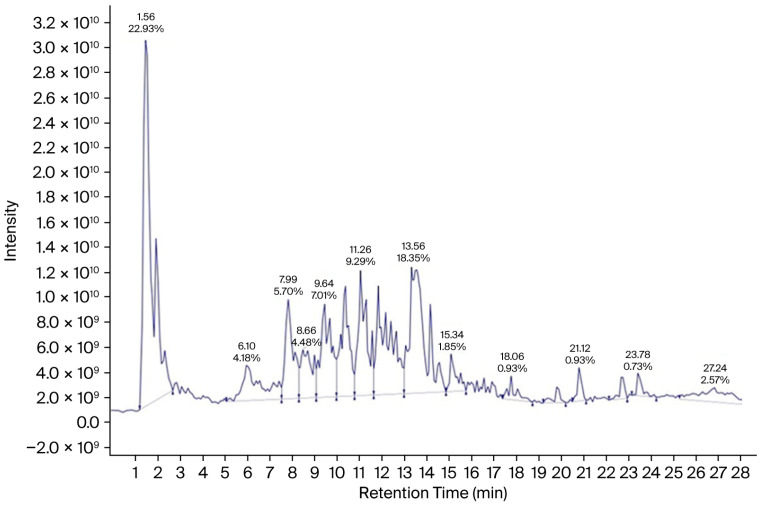
LC–MS/MS ESI-TIC chromatogram of ethyl acetate extract of *A. terreus* negative mode.

**Figure 3:**
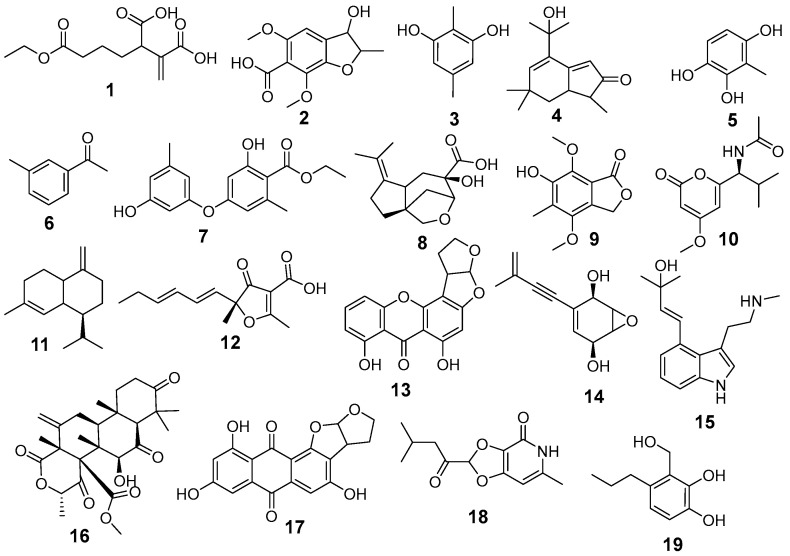
Chemical structures of identified compounds **1–19**.

**Figure 4:**
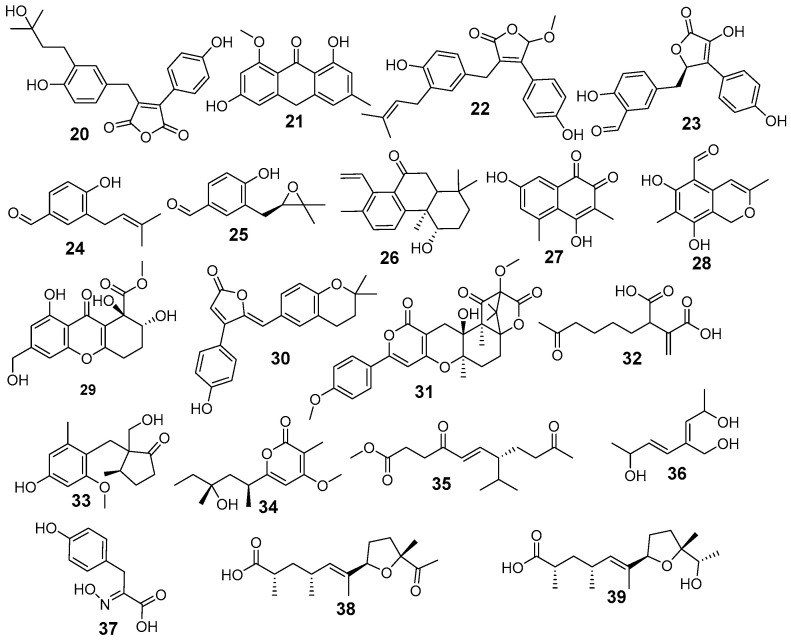
Chemical structures of identified compounds **20–39**.

LC-ESI-MS/MS analysis of *A. terreus* EtOAc extract was carried out for the separation and detection of secondary metabolites. Negative ionization mode was used to characterize the corresponding signals and the total ion current map of the extract was obtained. Figure 2 illustrates the TIC of *A. terreus* EtAOc extract. Structural and two-stage mass spectrometry analysis was employed to obtain the mass, characteristic fragmentations, and molecular formula of the components. Additionally, previous studies of isolated compounds from the *Aspergillus* genus and *A. terreus* were utilized as an identification tool of the detected compounds by comparing constituents’ characteristics with the published data. Analysis of the *A. terreus* extract revealed that several compounds (39) were proposed in negative ion mode, depending on their retention time, precursor ion, and MS2 fragmentation patterns compared with the Competitive Fragmentation Modeling for Metabolite Identification online database (Table 4, Figure 3 and Figure 4). The detected compounds were arranged according to their retention time.

The molecular ion mass peaked at m/z 253.06, 233.08, 251.11, 203.06, 473.24, 381.08, 379.15, 383.08, 189.06, 205.05, 347.07, 509.21, 277.15, and 267.17 [M − H]^−^, fitted with asperterreusine B, brasilanone A, aspterric acid, *γ*-cadinene, terreustoxin F, aperterone B, (±)-asperteretal D,3-hydroxy-4-(4-hydroxyphenyl)-5-methoxycarbonyl-5-(4-hydroxy-3-formylbenzyl)-2,5-dihydro-2-furanone, 4-hydroxy-3-(3-methylbut-2-enyl)benzaldehyde, terreprenphenol C, rubrolide S, terreulactone A, aspereusin C, and aspereusin E, respectively. Moreover, the mass ion peaks at Rt of 2.10, 3.33, 3.61, 6.16, 6.65, 7.12, 8.70, 8.75, 8.77, 10.67, 10.76, 10.84, 12.05, 14.71, 14.80, 15.24, 15.41, 18.65, 19.78, 23.40, 23.72, and 27.42 min gave hits for tensyuic acid F, 2,5-dimethylresorcinol, versicolin, 1-(3-Methylphenyl)-ethanone, ethericin B, 7-methoxyporriolide, asperfuranone B, dihydrodemethylsterigmatocystin, asperpentyn, isoversicolorin C, isoflavipucine, asperpanoid A, anhydroasperflavin, diterpenoid 6-deoxyaspergiloid C, aspergiodiquinone, anishidiol, aspergillusone B, asperitaconic acid C, nipyrone C, aspinotriol A/B, asperic acid A, and asperic acid B, respectively. The results of negative mode analysis showed the existence of nitrogen-containing compounds at Rt of 7.19, 9.07, and 23.68 min, corresponding to molecular formulae C_12_H_17_NO_4_, C_16_H_22_N_2_O, and C_9_H_9_NO_4_ assigned for campyrone C, ergotryptamine, and 4-hydroxyphenylpyruvic acid oxime, respectively (Supplementary Material; representative MS/MS spectra and additional raw data).

## 4. Discussion

Endophytic fungi are a source of a variety of secondary metabolites that are important but not extensively investigated, especially those associated with plants adapted to arid and semi-arid environments, such as date palms (*Phoenix dactylifera*). In this study, eleven fungal strains were isolated from Sukkari date fruits following plating on PDA and subsequent purification. Among them, *Aspergillus terreus* (SU-5) was selected for detailed analysis based on its metabolite profile and superior bioactivity, which aligns with previous reports highlighting its potential as a producer of bioactive secondary metabolites [9,10,11,12,13,14,15,16]. The chemical analysis of an endophytic *A. terreus* (SU-5) revealed a diverse range of known fungal metabolites, reflecting the high biosynthetic capacity of this species under endophytic conditions. Thirty-nine metabolites were detected, including polyketides, quinones, and phenolics. Although these compounds have previously been reported from various *A. terreus* strains [37,42,45,50,54,56,57,58,64,67], this is the first report of metabolites such as versicolin, isoversicolorin C, anhydroasperflavin, aspergiodiquinone, anishidiol, aspergillusone B, and nipyrone C from an endophytic *A. terreus* inhabiting date fruits [39,51,55,61,62,63,68]. One limitation of the present study is that fermentation was performed using a single substrate, which may influence the metabolic profile of the fungus. Previous studies have shown that variations in culture conditions and nutrient composition can significantly affect fungal secondary metabolite production and diversity [72,73]. Therefore, future studies should investigate the use of alternative substrates and optimized fermentation conditions to explore potential changes in metabolite profiles. Additionally, the LC–MS-based metabolite identification performed in this study provides only tentative assignments based on database comparisons. Consequently, further work should focus on the isolation and purification of the major bioactive compounds followed by structural elucidation using spectroscopic techniques such as NMR and HR-MS to confirm their identities [74]. The extract of *A. terreus* exhibited a substantial concentration of phenolic compounds (93.13 ± 1.57 mg GAE/g) and flavonoids (22.70 ± 0.57 mg QE/g) in comparison to previous research; Das et al. [75] indicated that the total phenolic content of extracts from eleven fungal endophytes derived from *Zingiber nimmonii* (J. Graham) Dalzell varied between 10.8 ± 0.7 and 81.6 ± 6.0 mg GAE/g dry extract. Flavonoids were detected in eight extracts, ranging from 5.2 ± 0.5 to 24.3 ± 0.9 mg QE/g of dry extract. *Bipolaris specifera* extracts had the highest total phenolic content (81.58 ± 6.0 mg GAE/g dry extract), followed by *A. terreus*. *Nectria hematococca* demonstrated elevated flavonoid content (24.3 ± 0.9 mg CE/g dry extract), but *A. terreus* displayed a lower concentration (11.3 ± 0.4 mg CE/g dry extract). In another investigation, Gautam et al. [76] found that the total phenolic content of the endophytic fungus *Nigrospora sphaerica*, isolated from the pantropical weed *Euphorbia hirta* L., was highest in EtOAcE fermented in potato dextrose broth (PDB) medium, measuring 77.74 ± 0.046 mg GAE/g. The significant production of phenolic compounds and flavonoids generated by *A. terreus* (SU-5) may result from its habitat of date fruits, which possess natural components that promote *A. terreus* metabolism, hence enabling the synthesis of phenolic and flavonoid compounds.

Interestingly, the crude extract of *Aspergillus terreus* showed a relatively high flavonoid content according to the aluminum chloride colorimetric assay; however, none of the metabolites identified by LC–MS analysis could be classified as flavonoids. This discrepancy may be attributed to the known limitations of the aluminum chloride method, which is not entirely specific for flavonoids [77,78]. Several non-flavonoid phenolic compounds can also form complexes with aluminum ions, leading to an overestimation of flavonoid content [77,79]. In the present extract, compounds such as anthraquinones, xanthones, and polyhydroxylated quinones contain keto-hydroxyl or ortho-dihydroxyl functional groups that may produce measurable responses in this assay, thereby contributing to the values expressed as quercetin equivalents (QE). Furthermore, LC–MS analysis allowed tentative identification of metabolites through comparison with available databases, and no flavonoids previously reported from fungi matched the observed molecular masses and fragmentation patterns. These results suggest that the predominant metabolites in the extract are more likely non-flavonoid polyketide derivatives rather than true flavonoids. Therefore, future studies will focus on the isolation of the major bioactive metabolites, followed by structural confirmation using NMR spectroscopy.

Sugars such as glucose, fructose, and sucrose constitute approximately 25–35% of the dry weight of dates and may serve as carbon sources that can be metabolized into intermediates such as acetyl-CoA and malonyl-CoA, which are commonly involved in polyketide biosynthesis. In addition, aromatic amino acids such as phenylalanine and tyrosine present in dates could potentially contribute to metabolic pathways related to phenolic compound formation through the shikimate pathway. Fatty acids, including oleic and palmitic acids, may also undergo catabolism to generate acetyl-CoA, which can serve as a precursor for polyketide chain elongation. However, these proposed links remain hypothetical and require further biochemical and metabolic investigations for confirmation [4,80]. The antioxidant activity of the EtOAc extract of *A. terreus* was determined using various in vitro assays: DPPH, ABTS radical scavenging, ferrous ion chelation, and peroxyl radicals in the ORAC assay. The extract exhibited variable antioxidative activity, with an IC_50_ value of 575.64 μg/mL for DPPH, 50.18 μg/mL for ABTS, 1007.82 μg/mL for ferrous ion chelation, and 3879.42 µmol TE/g against peroxyl radicals in the ORAC assay. All tests, in comparison to the standard antioxidants (BHT, Trolox, and EDTA), demonstrated elevated IC_50_ values, indicating diminished antioxidant efficacy. It is essential to emphasize that the study assessed crude fungal extract rather than purified components [75]. However, our study showed lower IC_50_ values when estimated with DPPH and ABTS assays in comparison to Zhou et al. [81], who reported that the fungal endophyte *Neopestalotiopsis protearum* exhibited an IC_50_ value of 1240 μg/mL when estimated with ABTS and 1800 μg/mL with the DPPH method. Another study by Elhosari et al. [82] indicated that the extract of *Aspergillus* sp. (TMP16) isolated from *Tabernaemontana pandacaqui* leaves showed considerable antioxidant capacity, measuring 881.49 ± 44.6 μM TE/mg with the DPPH assay and 866.86 ± 50.0 μM TE/mg through the ABTS assay. Five endophytic strains, namely *Aspergillus niger*, *Penicillium glabrum*, *Alternaria alternata*, *A. tenuissima*, and *Mucor circinelloides*, isolated from the stems of *Gundelia tournefortii*, were evaluated for antioxidant activity using the DPPH assay. The antioxidant activity of isolates indicated that *A. alternata* extract (IC_50_ = 471 ± 29 μg/mL) exhibited the most potent antioxidant activities, followed by *A. tenuissima* extract (IC_50_ = 512 ± 19 μg/mL), while the extracts of *M. circinelloides* and *A. niger* exhibited no antioxidant activities [83]. Das et al. [75] indicated that the extract of *B. specifera* exhibited elevated levels of total phenolic compounds and ABTS scavenging ability among all isolates, although minimal DPPH scavenging action was shown (1057.2 ± 122.3 μg/mL). Conversely, *A. tenuissima* had significant DPPH scavenging ability (96.9 ± 2.4 μg/mL) followed by *A. terreus* (IC_50_ 123.3 ± 7.6 μg/mL) and *N. hematococca* (IC50 133.4 ± 5.3 μg/mL), despite its total phenolic contents being lower than those of *B. specifera*. The numbers of hydroxyl moieties attached to the aromatic ring are favorable for the DPPH radical scavenging activity of phenolic acids. In our study, 13 phenolic compounds were found from a total of 39 compounds. Versicolin and isoversicolorin C possess three hydroxyl groups, whereas asperteretal D, 3-hydroxy-4-(4-hydroxyphenyl)-5-methoxycarbonyl-5-(4-hydroxy-3-formylbenzyl)-2,5-dihydro-2-furanone, and anishidiol includes two hydroxyl groups. The antioxidant efficacy of the extract is attributed to the significant presence of isoversicolorin C, as indicated by the peak area analysis. Yang et al. [51] isolated the mangrove-derived endophytic fungus *Aspergillus nidulans* MA-143, producing isoversicolorin C, under 0.1% ethanol stress, demonstrating antibacterial efficacy against Gram-negative bacteria such as *E. coli*, *M. luteus*, and *V. vulnificus*. In comparison to the assays that were tested, the ABTS assay demonstrated a high level of antioxidant activity. This was consistent with the fact that the majority of the phenolic compounds identified (versicolin, ethericin B, isoversicolorin C, anhydroasperflavin, aspergiodiquinone, aspereusin C, and aspereusin E) are polyphenolic, quinonoid, or polar. ABTS favors structures with more efficient electron transfer, which is poor for DPPH [84]. The fungal extract exhibited weak cytotoxic activity against MCF-7 cells (IC_50_ = 424.73 ± 8.29 μg/mL) and markedly reduced the activity against MCF-7/Adr cells (IC_50_ = 1140.77 ± 21.33 μg/mL), yielding a resistance index of 2.68. This differential response suggests limited effectiveness against multidrug-resistant cancer phenotypes and indicates that the cytotoxic constituents may not be substrates for resistance-associated transporters. The moderate activity observed is consistent with the presence of quinone- and polyketide-based metabolites such as aspergiodiquinone, isoversicolorin C, and aspergillusone B, which have previously been associated with antiproliferative effects in cancer cell models [85]. Although these results indicate potential antiproliferative properties, the observed activity should be interpreted cautiously. The potency of these metabolites remains moderate compared with clinically used anticancer agents, and further studies, including mechanistic investigations and in vivo validation, are required to determine their potential therapeutic relevance. In addition, the fungal extract exhibited weak inhibitory activity against acetylcholinesterase (IC_50_ = 456.90 µg/mL) and slightly stronger inhibition of butyrylcholinesterase (IC_50_ = 157.10 µg/mL), indicating a degree of selectivity toward BChE. This selectivity is pharmacologically relevant, as BChE activity is known to increase during the progression of Alzheimer’s disease. Phenolic and quinone-containing metabolites, including rubrolide S, anhydroasperflavin, aspergiodiquinone, and aspereusin derivatives, are likely contributors to this activity due to their ability to interact with the active and peripheral sites of cholinesterase enzymes through hydrogen bonding and π–π interactions [86]. However, the observed inhibition was moderate compared with standard cholinesterase inhibitors. Therefore, these findings should be considered preliminary, and further studies are required to clarify their mechanism of action and potential relevance for cholinesterase-related disorders.

## 5. Conclusions

For the first time, this study highlighted the potential role of the endophytic fungus *Aspergillus terreus* isolated in date fruits (*Phoenix dactylifera*) as a source of a diverse array of secondary metabolites including polyketides, quinones, and phenolics. The fungal extract of *A. terreus* exhibited a significant concentration of phenolic compounds that correlated with its antioxidant activity, moderate cytotoxic activity against MCF-7 cells, and inhibitory effects against AChE and BuChE enzymes. These findings support further research into the eco-friendly sourcing of antioxidants from endophytes residing in date fruits, potentially yielding sustainable alternatives to synthetic compounds. Metabolites of *A. terreus* showed multifunctional potential, integrating antioxidant and cytotoxicity effects, which could be advantageous for food preservation or pharmaceuticals.

## Figures and Tables

**Table 1:** Total phenolic and flavonoid content and antioxidant activities (IC_50_ values) of ethyl acetate extract from endophytic *A. terreus*.

Extract	Total Phenolics (mg GAE/g)	Total Flavonoids (mg QE/g)	DPPH IC_50_ (μg/mL)	ABTS IC_50_ (μg/mL)	Metal Chelation IC_50_ (μg/mL)	ORAC (µmol TE/g)
*A. terreus*	93.13 ± 1.57	22.70 ± 0.57	575.64 ± 3.24 ^b^	50.18 ± 0.11 ^b^	1007.82 ± 27.38 ^b^	3879.42 ± 138.12
BHT	-	-	38.07 ± 0.51 ^a^	-	-	-
Trolox	-	-	-	6.17 ± 0.09 ^a^	-	-
EDTA	-	-	-	-	12.45 ± 0.09 ^a^	-

-: Not tested. Data were presented as the mean of three replicates (mean ± SD). Values associated with superscripts differ significantly, with *p* < 0.05. a = control, b = variant.

**Table 2:** IC_50_ dose–response analysis for MCF-7 and MCF-7-Adr cell lines and brine shrimp lethality assay (LC_50_ value) using endophytic *A. terreus* extract.

Extract	MCF7 IC_50_ (μg/mL)	MCF-7-Adr IC_50_ (μg/mL)	LC_50_ (µg/mL)
*A. terreus*	424.73 ± 8.29 ^b^	1140.77 ± 21.33	1402.92 ± 33.58
Doxorubicin	19.78 ± 1.10 ^a^	NT	NT

NT: Not tested. Data were presented as the mean of three replicates (mean ± SD). Values associated with superscripts differ significantly, with *p* < 0.05. a = control, b = variant.

**Table 3:** Acetylcholine and butyrylcholine esterase inhibition (IC_50_ values) of endophytic *A. terreus* extract.

Extract	Acetylcholine Esterase Inhibition IC_50_ (μg/mL)	Butyrylcholine Esterase Inhibition IC_50_ (μg/mL)
*A. terreus*	456.90 ± 34.85 ^b^	157.10 ± 9.77 ^b^
Donepezil	0.0002632 ± 0.0000019 ^a^	0.2418 ± 0.032 ^a^

Data were presented as the mean of three replicates (mean ± SD). Values associated with superscripts differ significantly, with *p* < 0.05. a = control, b = variant.

**Table 4:** List of compounds tentatively identified by LC-MS/MS analysis for *A. terreus* extract.

No.	Compound	Molecular Formula	Compound	Mass	[M − H]^−^	Area %	MS2 Fragments	Source	Ref.
1	Tensyuic acid F	C_11_H_16_O_6_	2.10	244.09	243.05	0.11	199, 197, 171, 157, 153	*Aspergillus niger* FKI-2342	[36]
2	Asperterreusine B	C_12_H_14_O_6_	Asperterreusine B	254.08	253.06	0.15	251, 221, 209, 197, 193, 181, 179, 165, 153	*Aspergillus terreus* [CFCC 81836]	[37]
3	2,5-Dimethylresorcinol	C_8_H_10_O_2_	2,5-Dimethylresorcinol	138.07	137.01	0.37	109, 119	*Aspergillus nidulans*	[38]
4	Brasilanone A	C_15_H_22_O_2_	Brasilanone A	234.16	233.08	0.17	217, 215, 175, 173, 159, 149, 147	*Aspergillus terreus* [CFCC 81836]	[37]
5	Versicolin	C_7_H_8_O_3_	Versicolin	140.05	139.01	1.02	111, 121	*Aspergillus versicolor*	[39]
6	1-(3-Methylphenyl)-ethanone	C_9_H_10_O	1-(3-Methylphenyl)-ethanone	134.07	133.03	0.09	99, 115, 117	*Aspergillus candidus*	[40]
7	Ethericin B	C_17_H_18_O_5_	Ethericin B	302.12	301.11	0.24	273, 257, 255, 229, 193	*Aspergillus funiculosus* Tü 680	[41]
8	Aspterric acid	C_14_H_20_O_4_	Aspterric acid	252.14	251.11	0.03	221, 219, 207, 205, 191, 189, 177, 175, 163	*Aspergillus terreus*	[42]
9	7-Methoxyporriolide	C_11_H_12_O_5_	7-methoxyporriolide	224.07	223.06	0.09	221, 207, 189, 179,161	*Aspergillus nidulans*	[43]
10	Campyrone C	C_12_H_17_NO_4_	Campyrone C	239.12	238.05	0.46	222, 194, 192, 180, 164, 152	*Aspergillus niger*	[44]
11	γ-Cadinene	C_15_H_24_	γ-Cadinene	204.19	203.06	0.30	187, 173, 157, 147, 121	*Aspergillus terreus*	[45]
12	Asperfuranone B	C_13_H_16_O_4_	Asperfuranone B	236.10	235.05	0.30	217, 205, 193, 191, 189, 175, 163, 155, 137	*Aspergillus* sp.	[46]
13	Dihydrodemethylsterigmatocystin	C_17_H_12_O_6_	Dihydrodemethylsterigmatocystin	312.06	311.08	2.95	309, 293, 281, 267, 255, 251, 243, 227	*Aspergillus* sp.	[47]
14	Asperpentyn	C_11_H_12_O_3_	Asperpentyn	192.08	191.07	2.31	189,175, 173,133,119,103	*Aspergillus duricaulis*	[48]
15	Ergotryptamine	C_16_H_22_N_2_O	Ergotryptamine	258.17	257.02	0.22	228, 224, 212, 210, 200, 198, 183, 168, 166, 140, 116	*Aspergillus nidulans*	[49]
16	Terreustoxin F	C_26_H_34_O_8_	Terreustoxin F	474.23	473.24	1.57	455, 425, 417, 399	*Aspergillus terreus*	[50]
17	Isoversicolorin C	C_18_H_12_O_7_	Isoversicolorin C	340.06	339.08	32.34	337, 323, 321, 311, 295, 271	*Aspergillus nidulans*	[51]
18	Isoflavipucine	C_12_H_15_NO_4_	Isoflavipucine	237.10	236.12	28.47	218, 208, 167, 152, 138, 124	*Aspergillus flavipes*	[52]
19	Asperpanoid A	C_10_H_14_O_3_	Asperpanoid A	182.09	181.02	0.17	163, 151, 137, 135, 123	*Aspergillus* sp. ZJ-68	[53]
20	Asperterone B	C_22_H_22_O_6_	Asperterone B	382.14	381.08	0.15	379, 363, 353, 351, 345, 335, 323, 309, 307, 305, 251, 201	*Aspergillus terreus* MHL-P22	[54]
21	Anhydroasperflavin	C_16_H_14_O_4_	Anhydroasperflavin	270.09	269.01	0.03	253, 239, 229, 227, 225, 223, 213, 197, 133	*Aspergillus flavus*	[55]
22	(±)-Asperteretal D	C_23_H_24_O_5_	(±)-asperteretal D	380.16	379.15	0.05	377, 362, 351, 347, 337, 323, 289	*Aspergillus terreus*	[56]
23	3-Hydroxy-4-(4-hydroxyphenyl)-5-methoxycarbonyl-5-(4-hydroxy-3-formylbenzyl)-2,5-dihydro-2-furanone	C_20_H_16_O_8_	3-hydroxy-4-(4-hydroxyphenyl)-5-methoxycarbonyl-5-(4-hydroxy-3-formylbenzyl)-2,5-dihydro-2-furanone	384.08	383.08	0.45	355, 351, 339, 323, 307, 297, 295, 203, 189	*Aspergillus terreus* var. *boedijnii* (Blochwitz)	[57]
24	4-Hydroxy-3-(3-methylbut-2-enyl)benzaldehyde	C_12_H_14_O_2_	4-Hydroxy-3-(3-methylbut-2-enyl)benzaldehyde	190.10	189.06	0.57	171, 161, 159, 143, 133, 121	*Aspergillus terreus* BCC51799	[58]
25	Terreprenphenol C	C_12_H_14_O_3_	Terreprenphenol C	206.09	205.05	0.03	203, 187, 177, 173,161,151, 147, 145, 135, 133	*Aspergillus terreus* EN-539	[59]
26	6-Deoxyaspergiloid C	C_20_H_26_O_2_	6-deoxyaspergiloid C	298.19	297.17	0.03	295, 279, 267, 253, 217, 197	*Aspergillus candidus*	[60]
27	Aspergiodiquinone	C_12_H_10_O_4_	Aspergiodiquinone	218.06	217.05	0.04	201, 189, 175, 173, 135	*Aspergillus glaucus* HB1-19	[61]
28	Anishidiol	C_12_H_12_O_4_	Anishidiol	220.07	219.06	0.06	217, 203, 191, 175, 173, 147	*Aspergillus nishimurae* IFM58441	[62]
29	Aspergillusone B	C_16_H_16_O_8_	Aspergillusone B	336.08	335.09	0.19	317, 305, 301, 289, 287, 265, 257, 245, 217, 203	*Aspergillus sydowii* PSU-F154	[63]
30	Rubrolide S	C_22_H_20_O_4_	Rubrolide S	348.14	347.07	10.79	331, 329, 319, 313, 311, 303, 291, 289, 277, 211	*Aspergillus terreus* OUCMDZ-1925	[64]
31	Terreulactone A	C_28_H_30_O_9_	Terreulactone A	510.19	509.21	0.19	481, 463, 453, 437, 415, 377	*Aspergillus terreus*	[65]
32	Asperitaconic acid C	C_11_H_16_O_5_	Asperitaconic acid C	228.10	227.15	0.23	209, 183, 167, 165, 163, 139, 137	*Aspergillus niger*	[66]
33	Aspereusin C	C_16_H_22_O_4_	Aspereusin C	278.15	277.15	0.04	275, 247, 233, 231, 221, 215, 193, 191	*Aspergillus terreus* YIM PH30711	[67]
34	Nipyrone C	C_14_H_22_O_4_	Nipyrone C	254.15	253.16	0.06	235, 207, 181, 167, 151, 135, 123	*Aspergillus niger*	[68]
35	Aspereusin E	C_15_H_24_O_4_	Aspereusin E	268.17	267.17	0.12	251, 249, 207, 195, 179	*Aspergillus terreus* YIM PH30711	[67]
36	Aspinotriol A/B	C_9_H_16_O_3_	Aspinotriol A/B	172.11	171.09	0.32	153, 135, 109	*Aspergillus ostianus*	[69]
37	4-Hydroxyphenylpyruvic acid oxime	C_9_H_9_NO_4_	4-Hydroxyphenylpyruvic acid oxime	195.05	194.05	0.08	176,161, 150, 148, 132, 119, 117	*Aspergillus aculeatus* CRI323-04	[70]
38	Aspericacid A	C_16_H_26_O_4_	Aspericacid A	282.18	281.17	0.07	263, 211, 209, 207	*Aspergillus* sp. LS78	[71]
39	Aspericacid B	C_16_H_28_O_4_	Aspericacid B	284.20	283.19	0.15	265,263, 239, 221, 211, 195, 193	*Aspergillus* sp. LS78	[71]

## Data Availability

Data of the measurement results are available from the authors.

## Supplementary Materials

The following supporting information can be downloaded at: https://www.mdpi.com/article/10.3390/jof12040249/s1, 1. Raw data 2. Representative MS/MS spectra for the major identified metabolites (e.g., Isoversicolorin C, Isoflavipucine, and Rubrolide S). 3. The contig sequences used for phylogenetic analysis (Table S1).

## Abbreviations

The following abbreviations are used in this manuscript:

A.	Aspergillus
AAPH	2,2′-Azobis(2-amidinopropane) dihydrochloride
ABTS	2,2′-azino-bis(3-ethylbenzothiazoline-6-sulfonic acid)
AChE	Acetylcholinesterase
BHT	Butylated hydroxytoluene
BSCT	Brine shrimp cytotoxicity test
BuChE	Butyrylcholinesterase
CE/g	Catechin equivalent per gram
DPPH	2,2-diphenyl-1-picrylhydrazyl
DMEM	Dulbecco’s Modified Eagle’s Medium
DMSO	Dimethyl sulfoxide
DTNB	5,5′-dithio-bis-(2-nitrobenzoic acid)
EDTA	Ethylenediaminetetraacetic Acid
EtOAcE	Ethyl acetate
GAE/g	Gallic acid equivalent per gram
IC_50_	Half-maximal inhibitory concentration
LC/MS/MS	Liquid chromatography-mass spectrometry analysis
MCF-7	Breast Adenocarcinoma
MCF-7-Adr	Doxorubicin-resistant Breast Cancer
ORAC	Oxygen Radical Absorbance Capacity
PDA	Potato Dextrose Agar medium
QE/g	Quercetin equivalents per gram
SRB	Sulforhodamine B
TCA	Trichloroacetic acid
TE/g	Trolox equivalents per gram
TIC	Total ion chromatogram
PCR	Polymerase chain reaction
