# Supplementary material for: Chemical and Biological Investigation of the Endophytic Aspergillus terreus (SU5) Inhabiting Date Fruits (Phoenix dactylifera)
Source: J Fungi (Basel). 2026 Mar 30;12(4):249. doi: 10.3390/jof12040249 (PMC13117145; doi:10.3390/jof12040249)
Supplement: Supplementary file 1 [file jof-12-00249-s001.zip › Representative MS spectra.pdf]

Rubrolide S	C22H20 O4	16.01	348.14	347.07	10.79	331, 329, 319, 313, 311, 303, 291, 289, 277, 211	*Aspergillus terreus* OUCMDZ-1925
Isoversicolorin C	C18H12 O7	10.67	340.06	339.08	32.34	337, 323, 321, 311, 295, 271	*Aspergillus nidulans*
Isoflavipucine	C12H15 NO4	10.76	237.10	236.12	28.47	218, 208, 167, 152, 138, 124	*Aspergillus flavipes*

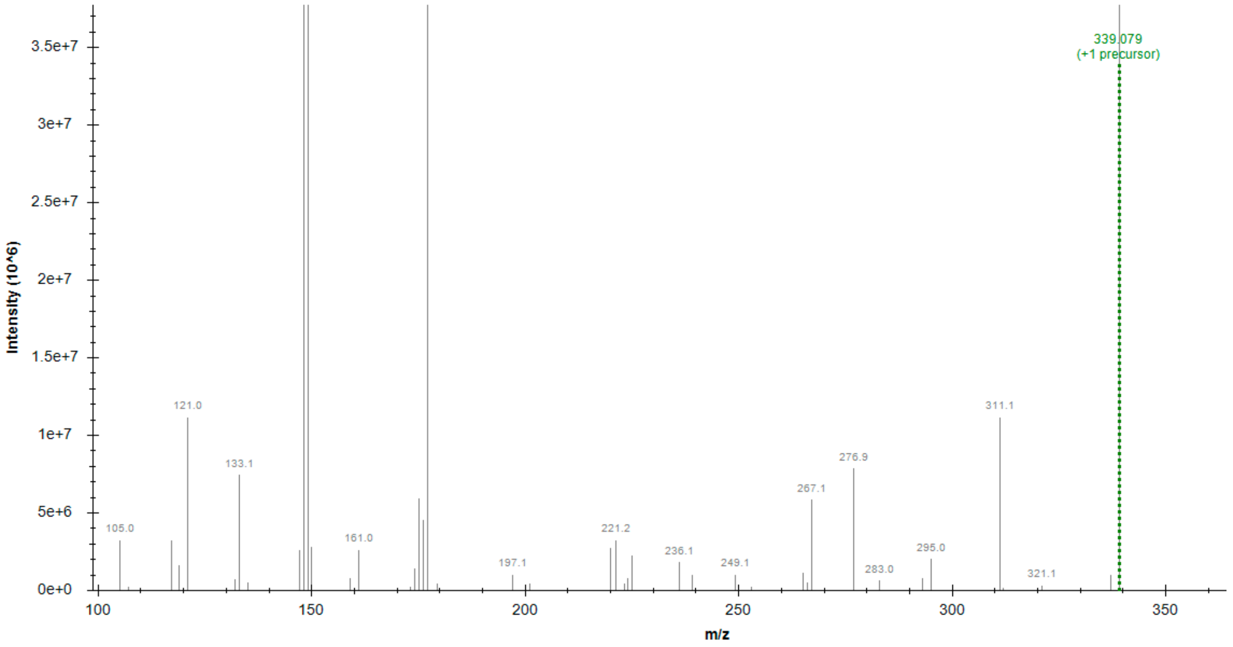

Mass spectrum of Isoversicolorin C

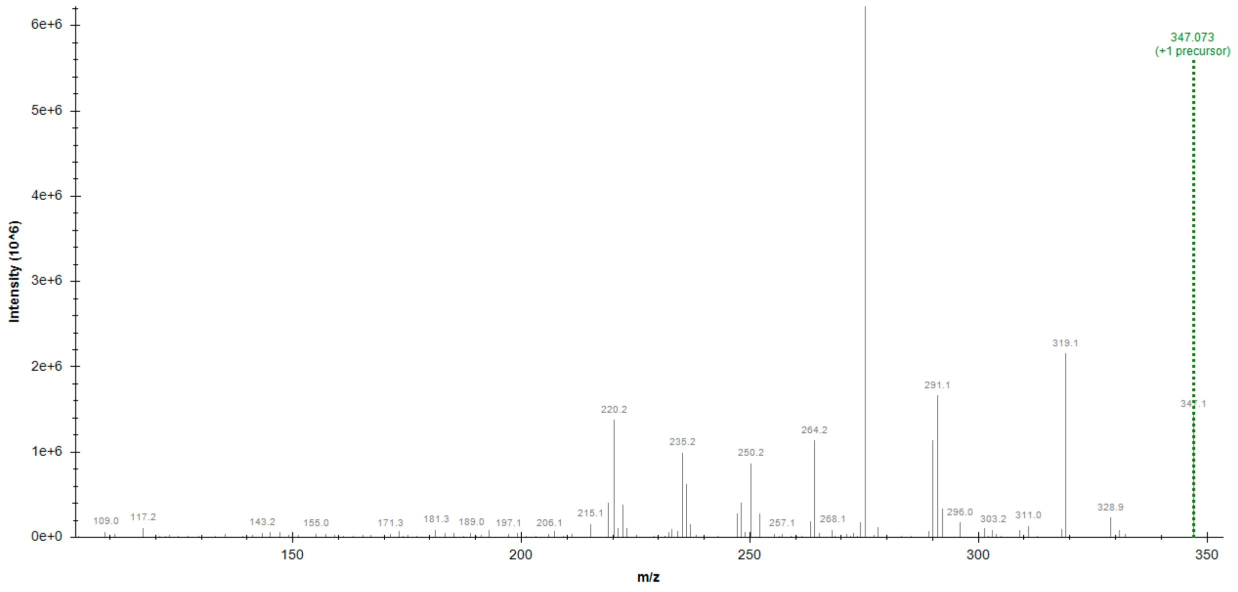

Mass spectrum of Rubrolide S

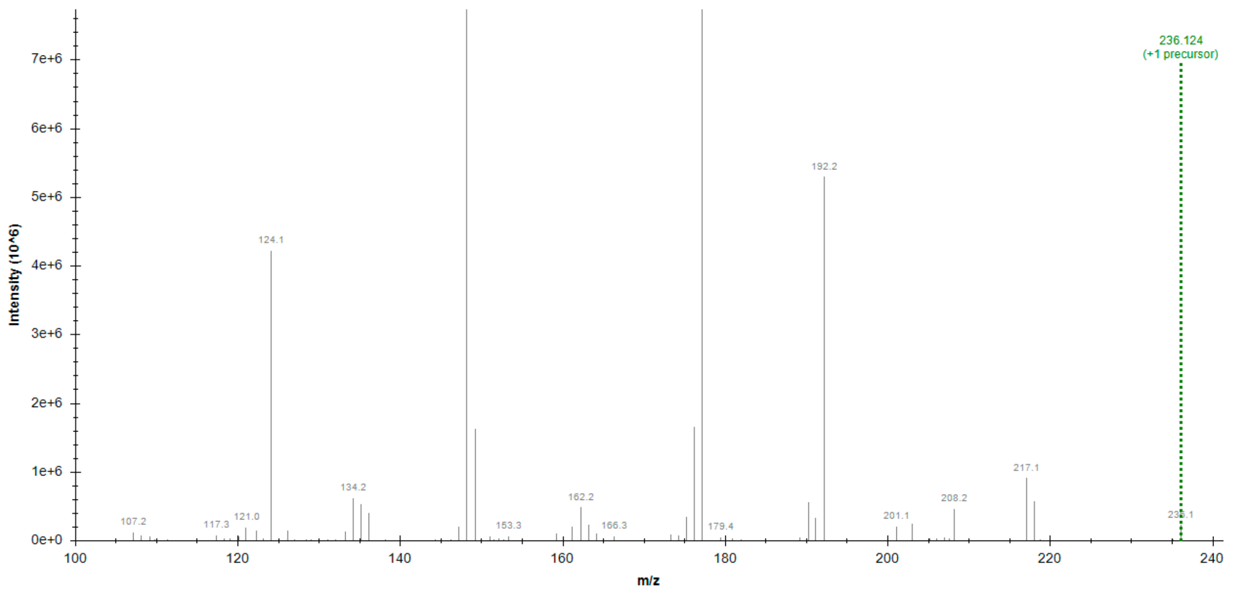

Mass spectrum of Isoflavipucine
